# Response to Treatment in a Prospective Cohort of Patients with Large Ulcerated Lesions Suspected to Be Buruli Ulcer (*Mycobacterium ulcerans* Disease)

**DOI:** 10.1371/journal.pntd.0000736

**Published:** 2010-07-06

**Authors:** Kapay Kibadi, Marleen Boelaert, Alexandra G. Fraga, Makanzu Kayinua, Adhemar Longatto-Filho, Jean-Bedel Minuku, Jean-Baptiste Mputu-Yamba, Jean-Jacques Muyembe-Tamfum, Jorge Pedrosa, Jean-Jacques Roux, Wayne M. Meyers, Françoise Portaels

**Affiliations:** 1 Programme National de Lutte contre l'Ulcère de Buruli (PNLUB), Kinshasa, Democratic Republic of the Congo; 2 Institut National de Recherche Biomédicale, Kinshasa, Democratic Republic of the Congo; 3 Department of Surgery, University of Kinshasa, Kinshasa, Democratic Republic of the Congo; 4 Epidemiology Unit, Institute of Tropical Medicine, Antwerp, Belgium; 5 Life and Health Sciences Research Institute (ICVS), School of Health Sciences, University of Minho, Braga, Portugal; 6 General Reference Hospital of Nsona-Mpangu, Lower-Congo, Democratic Republic of the Congo; 7 Rural Health Zone of Nsona-Mpangu, Lower-Congo, Democratic Republic of the Congo; 8 Laboratory of Medical Investigation (LIM) 14, Department of Pathology of Faculty of Medicine of São Paulo State University, São Paulo, Brazil; 9 Anatomy Pathology Unit, Hospital of Chambéry, Association Pathologie Cytologie Développement, Chambéry, France; 10 Armed Forces Institute of Pathology (AFIP), Washington, D.C., United States of America; 11 Mycobacteriology Unit, Institute of Tropical Medicine, Antwerp, Belgium; Kwame Nkrumah University of Science and Technology (KNUST) School of Medical Sciences, Ghana

## Abstract

**Background:**

The World Health Organization (WHO) advises treatment of *Mycobacterium ulcerans* disease, also called “Buruli ulcer” (BU), with a combination of the antibiotics rifampicin and streptomycin (R+S), whether followed by surgery or not. In endemic areas, a clinical case definition is recommended. We evaluated the effectiveness of this strategy in a series of patients with large ulcers of ≥10 cm in longest diameter in a rural health zone of the Democratic Republic of Congo (DRC).

**Methods:**

A cohort of 92 patients with large ulcerated lesions suspected to be BU was enrolled between October 2006 and September 2007 and treated according to WHO recommendations. The following microbiologic data were obtained: Ziehl-Neelsen (ZN) stained smear, culture and PCR. Histopathology was performed on a sub-sample. Directly observed treatment with R+S was administered daily for 12 weeks and surgery was performed after 4 weeks. Patients were followed up for two years after treatment.

**Findings:**

Out of 92 treated patients, 61 tested positive for *M. ulcerans* by PCR. PCR negative patients had better clinical improvement than PCR positive patients after 4 weeks of antibiotics (54.8% versus 14.8%). For PCR positive patients, the outcome after 4 weeks of antibiotic treatment was related to the ZN positivity at the start. Deterioration of the ulcers was observed in 87.8% (36/41) of the ZN positive and in 12.2% (5/41) of the ZN negative patients. Deterioration due to paradoxical reaction seemed unlikely. After surgery and an additional 8 weeks of antibiotics, 98.4% of PCR positive patients and 83.3% of PCR negative patients were considered cured. The overall recurrence rate was very low (1.1%).

**Interpretation:**

Positive predictive value of the WHO clinical case definition was low. Low relapse rate confirms the efficacy of antibiotics. However, the need for and the best time for surgery for large Buruli ulcers requires clarification. We recommend confirmation by ZN stain at the rural health centers, since surgical intervention without delay may be necessary on the ZN positive cases to avoid progression of the disease. PCR negative patients were most likely not BU cases. Correct diagnosis and specific management of these non-BU ulcers cases are urgently needed.

## Author Summary

Buruli ulcer (BU) disease, a neglected devastating infection caused by *Mycobacterium ulcerans*, has a huge impact because of the massive necrotizing, disfiguring ulcers that may result if not treated. Therapeutic options are surgery, antibiotics or combinations of both. Since 2004, the World Health Organization has recommended the use of antibiotics (rifampicin and streptomycin) for the management of the disease. The effectiveness of this antibiotic treatment on advanced lesions is, however, not well documented. We evaluated this strategy on large ulcers clinically suspected to be BU, in a rural zone of the Democratic Republic of Congo, and also assessed the outcome of treatment based only on clinical diagnosis. All patients were treated with antibiotics for 12 weeks and surgery was performed after 4 weeks. BU was confirmed by laboratory tests in 67% of the patients indicating that the clinical diagnosis of ulcerated forms of BU may be more difficult than usually reported. Although delayed surgery seemed detrimental in some confirmed cases, it was possible to treat 92% of patients successfully with low recurrence rates (1.1%) by combining antibiotic treatment with surgery in a rural zone. However, the need for and the best time for surgery for large Buruli ulcers requires clarification.

## Introduction

*Mycobacterium ulcerans* disease, commonly called “Buruli ulcer” (BU), is a neglected and emergent tropical disease [1], [2], with Africa being the most affected continent [3]. For many years, management of the disease relied mainly on surgical procedures [4], [5], [6]. Other treatment strategies included antibiotics alone or followed by surgery [7], [8].

A proof-of-principle study (phase-2 trial) conducted in Ghana evaluated the efficacy of the combination of rifampicin and streptomycin (R+S) on early BU lesions (nodules and plaques), and found that after 4 weeks of treatment with these drugs, it was no longer possible to cultivate *M. ulcerans* from these lesions [9]. This pilot trial led to the World Health Organization (WHO) recommendation to treat all BU lesions with R+S, whether followed by surgery or not [10].

WHO guidelines define three categories of treatment based on: (1) clinical form (ulcerative or non-ulcerative), (2) lesion size (lesions less than 5 cm and lesions of 5 cm or more in diameter), and (3) disseminated or mixed forms. Antibiotic treatment of 8 weeks is recommended for all three categories. For lesions ≥5 cm, surgery is recommended, if necessary, after at least 4 weeks of antibiotic treatment [10]. In 2005, WHO indicated that for very large lesions, antibiotic treatment may be administered for up to 12 weeks [11].

A case-series in Benin showed that of 224 patients treated by the WHO strategy, 215 were successfully treated, with 47% of them receiving antibiotics only. The size of the lesion was the major parameter in deciding to treat by surgery: 73% of patients with lesions of >15 cm in diameter underwent surgery, compared to 17% of patients with lesions of <5 cm [12]. More recently, Nienhuis et al. demonstrated that antimycobacterial treatment alone was effective in 151 patients with early limited BU disease [13]. The efficacy of R+S therapy on large ulcerated forms of BU, which currently are the most common forms of BU in Africa [14], [15], [16] remains insufficiently documented. In such cases the efficacy of antibiotics could be compromised by the extent of the necrosis [17].

The objective of the present study was to estimate the efficacy of the standard WHO recommended regimen (R+S followed by surgery) in patients with large ulcerated ulcers (≥10 cm in longest diameter) in a rural health zone (RHZ) of the Democratic Republic of Congo (DRC). The data obtained allowed us to assess the positive predictive value of the WHO clinical case definition for BU.

## Materials and Methods

### Study design

**Figure 1:**
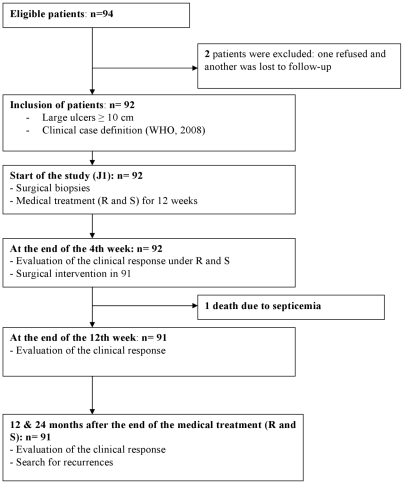
Flow sheet showing process of patient selection and management.

This is a prospective observational study that analyzes the response of patients treated with R+S for 12 weeks and usually followed by surgery after the first 4 weeks, a slight adaptation from the 2005 WHO protocol [11]. Study procedures are summarized in the flow sheet (Figure 1).

### Study population

Patients clinically suspected of BU were recruited from the RHZ of Nsona-Mpangu, Province of Lower-Congo in DRC. This health zone has long been known to be moderately endemic for BU [15], [18]. Patients were enrolled between October 2006 and September 2007 in 17 Health Centers and one General Reference Hospital. Suspected cases were identified by the head nurse of the Health Centre and later confirmed by two physicians, both co-authors, (MK, Supervisor of the BU Control Program in the RHZ of Nsona-Mpangu and J-BM, Chief of the RHZ of Nsona-Mpangu). After this, the Principal Investigator (PI) (KK), personally reviewed all cases. The final decision on classification of cases for this study was reached by consensus of the three physicians (KK, MK and J-BM).

For quality assurance, we conducted a post-hoc confirmation of the clinical classification by evaluating photographs of lesions taken at the start of treatment, after 4 weeks of antibiotic treatment, at the end of the antibiotic treatment and at the healed stage. A sample of these photographs was checked retrospectively by three individuals: two other co-authors (WMM and FP) and by an African colleague (Dr. G. Sopoh) from Benin all of whom are experienced in the clinical diagnosis of BU.

### Case definition

The clinical case definition of BU in this study was, “*Ulcerative lesions (maximum diameter ≥10 cm), painless or minimally painful, with characteristic undermined edges and a yellowish-white necrotic base surrounded by edematous skin*”. All consenting patients fitting this definition were treated in accordance with WHO recommendations. They were assessed by the PI to determine inclusion in the study. Biological samples were simultaneously collected and sent to reference laboratories for case confirmation by 4 laboratory methods: Ziehl-Neelsen (ZN) staining, culture, PCR, and histopathology [1].

### Inclusion criteria

1. Male and female, 3 to 75 years old
2. Residence in an endemic area (RHZ Nsona-Mpangu)
3. Informed consent by patient or guardian

### Exclusion criteria

1. Previous treatment by rifampicin or streptomycin
2. Previous diagnosis of leprosy or tuberculosis
3. Pregnancy
4. Presence of cardiovascular, hepatic or renal disease, detected during a complete physical examination
5. Loss to follow-up

### Procedures

#### Size of lesions, collection of specimens

We used the following methods to document the size of the lesions before, during and after treatment. Size of lesion was calculated by measuring the average diameter of the ulcer, taken as the mean of the longest (at least 10 cm) and shortest perpendicular diameters. Since the limit of the skin lesion was usually larger than the ulcer edges, diameters were measured from the border between healthy skin and damaged skin. Tissue samples were taken under local or general anesthesia at the start of treatment. Specimens from the undermined edges were taken only of diseased tissue.

#### Laboratory analyses

Biopsy specimens were stored at 4°C and transported at ambient temperature in a semi-solid transport medium to the Institute of Tropical Medicine (ITM), Antwerp, Belgium [19], where ZN staining, culture and PCR were carried out as previously described [20]. Histopathologic analyses were performed at three laboratories: Armed Forces Institute of Pathology (AFIP), Washington DC, USA; Hospital of Chambéry, France; and University of Minho, Braga, Portugal. At the time of this study, due to logistical constraints for transport of biological samples from this remote rural area of the DRC and communication difficulties, the delay between enrollment and availability of PCR results at point of care was at least 12 weeks.

#### Treatment regimen

All patients who satisfied the WHO clinical case definition were treated according to WHO recommendations [10], [11]: i.e. 4 weeks of antibiotic treatment (R+S), followed by surgery after day 28, and followed by 8 more weeks of R+S. Rifampicin was administered orally at 10 mg/kg/day and streptomycin by intramuscular injection at 15 mg/kg/day. Surgical excision and skin grafting of the ulcers were carried out according to procedures recommended by the WHO [5]. Dressings were changed daily with an aqueous solution of chloramine-metronidazole-nitrofurandoïne [6].

#### Treatment assessment

Ambulatory follow-up for all patients was performed during treatment, except for those who lived far from the Health Centre, had severe lesions, or had just undergone surgery and were still hospitalized in the Health Centre.

A first evaluation was carried out at the end of the 4th week of R+S. The clinical outcome was classified as **success** (10 to 30% reduction of the size of the ulcer and/or absence of new necrotic tissue), **clinical status quo** (no change in the size of the lesion or presence of necrotic tissue), or **failure** (increase in the size of the lesion and presence of new necrotic tissue). The same evaluation was carried out at the end of the 12th week of treatment, the only difference being that success was interpreted as healed lesions.

#### Post-treatment assessment

For follow-up, patients were seen in their village every 2 weeks by the head nurse, and every month by the PI and the program supervisor. Disease recurrence was defined as the reappearance of an ulcer or another form (nodule, papule, plaque, edema or bone involvement) of the disease at the original site of the lesion or elsewhere during the 12 months that followed the end of antibiotic treatment. Follow-up after completion of treatment was at least 2 years for all patients.

#### Sample size considerations

Based on the assumptions that the true efficacy rate (p) was 80% and with a desired precision of 10%, for a confidence interval of 95%, we needed a sample size of 64 patients.

#### Data analysis

All clinical and laboratory data were entered into a patient Case Record Form. The data were further entered into an EPIINFO (Center for Disease Control and Prevention, Atlanta, USA) database and analyzed by EPIINFO and SPSS version 15.0 (Chicago, IL, USA) for Windows. Contingency tables were analyzed by the Pearson chi-square test and Fisher exact tests with a statistical significance-level of α = 0. 05.

### Ethical aspects

The study protocol was approved by the Ethical Committees of the Universitair Ziekenhuis Antwerpen, (N° 6/42/197) in Belgium and of the School of Public Health, University of Kinshasa, (N° ESP/CE/043) in DRC. Management of patients was free of charge. Participation in the survey was voluntary and written informed consent was obtained from all participants or their guardian.

## Results

Of 94 eligible patients, two were excluded: one refused treatment and one was lost to follow-up. Overall, 92 patients were included in this study with a mean ulcer size of 13.81 cm (SD 16.21). The male/female ratio was 0.88. Only patients with ulcers of more than 10 cm in longest diameter were included, thus 90 patients were classified as WHO Category II and two as Category III because of multiple lesions (Table 1).

**Table 1:** Characteristics of PCR positive and PCR negative patients at start of treatment.

	PCR positive patients (group I)	PCR negative patients (group II)	P value
	n = 61	n = 31	
Age (year)			p = 0.398
<15	28 (45.9%)	10 (32.2%)	
15–49	27 (44.3%)	16 (51.6%)	
≥50	6 (9.8%)	5 (16.1%)	
Gender			p = 0.046
Female	37 (60.6%)	12 (38.7%)	
Male	24 (39.3%)	19 (61.2%)	
Localization			p = 0.168
Trunk	6 (9.8%)	4 (12.9%)	
Buttock	1 (1.6%)	-	
Upper limb	20 (32.8%)	4 (12.9%)	
Lower limb	32 (52.5%)	23 (74.2%)	
Multiple	2 (3.3%)	-	
Ulcer size			
Average diameter cm (SD)	10.07 (1.95)	11.39 (5.82)	p = 0.320
Laboratory tests			
Direct microscopy			p<0.001
Positive ZN	48 (78.7%)	0 (0%)	
Negative ZN	13 (21.3%)	31 (100%)	
Culture			p<0.001
Positive	22 (36.1%)	0 (0%)	
Negative	39 (63.9%)	31 (100%)	
Histopathologic features (49 patients)			p<0.001
Compatible with BU	19/20 (95.0%)	4/29 (13.8%)	
Not compatible	1/20 (5.0%)	25/29 (86.2%)	

A total of 61 (66.3%) patients were positive by PCR for *M. ulcerans* (group I) and 31 were PCR negative (group II) at the start of treatment. Table 1 presents the characteristics of group I and group II patients at start of treatment. There was no age difference noted between the two groups. There were significantly more female patients in group I (60.6%) compared to group II (38.7%) (p = 0.046). The lower limbs were significantly more frequently affected in group II (74.1%) than in group I (52.4%) (p = 0.044). The average diameter of the ulcer in group I was 10.07 cm (SD = 1.95). In group II, it was 11.39 cm (SD 5.82). The difference in initial diameter of the ulcers between groups I and II at the beginning of treatment was not statistically significant (p = 0.320).

Of the 61 PCR positive patients (group I), 48 patients (78.7%) had a positive ZN and 22 (36.1%) were culture positive for *M. ulcerans*, whereas none (0%) of the 31 PCR negative (group II) patients were ZN positive or culture positive (p<0.001).

Histopathologic data were available for 49 patient samples (20 PCR positive and 29 PCR negative). As shown in Table 1, 95.0% (19/20) of the PCR positive patients had histopathologic features compatible with BU: i.e. contiguous coagulation necrosis of the lower dermis, subcutaneous tissue and underlying fascia; vasculitis in the subcutaneous tissue, and presence of AFB. None of the 29 PCR negative patient samples were positive for AFB. In 25 patients (86.2%) histopathologic analysis revealed no characteristic feature of BU and specimens from 12 patients (48.0%) showed chronic inflammation. Five patients (20.0%) had bacterial suprainfections with gram-positive cocci. Four patients (16.0%) had other dermatologic affections (impetigo, pyogranulomatous dermatitis, chronic dermatitis, hidradenitis). Three patients had vascular disorders and one a dermatophytosis. Only four of the 29 PCR negative patients had histopathologic changes compatible with BU but were AFB negative.

Clinical responses in the 2 groups after 4 weeks of antibiotic treatment are presented in Table 2. PCR negative patients had a higher percentage of clinical improvement (54.8%) than PCR positive patients (14.8%). This difference was statistically significant (p<0.001). As shown in Table 3, for the 61 PCR-positive patients, the clinical outcome at the 4th week assessment was related to ZN and culture results at the start of treatment. Indeed, after 4 weeks of R+S, significantly more treatment failures were observed among the ZN positive patients, 75.0% (36/48), compared to only 38.5% (5/13) of the ZN negative patients (p = 0.013). Treatment successes were obtained in 6.3% (3/48) of the ZN positive patients compared to 46.2% (6/13) of the ZN negative patients. Similarly the outcome was more successful for culture negative patients (8/39 or 20.5%) than for patients with positive *M. ulcerans* cultures (1/22 or 4.5%). The latter difference, however, was not significant.

**Table 2:** Response to the antibiotic combination, rifampicin + streptomycin followed by surgery, in patients with a clinical diagnosis of Buruli ulcer, according to initial PCR status.

	PCR positive patients (group I)	PCR negative patients (group II)	P value
Clinical outcome at 4 week assessment (before surgery)	n = 61	n = 31	p<0.001
Success	9 (14.8%)	17 (54.8%)	
Status quo	11 (18.0%)	2 (6.5%)	
Failure	41 (67.2%)	12 (38.7%)	
Clinical outcome at 12 week assessment (R&S with surgery)	n = 61	n = 30	p = 0.023
Success	60 (98.4%)	25 (83.3%)	
Status quo	0	1 (3.3%)	
Failure	1 (1.6%)	4 (13.3%)	
Average time of scarring (weeks)	10.4	7.5	
Recurrences	1	0	

**Table 3:** Clinical outcome of 61 PCR-positive Buruli ulcer patients after 4 weeks of antibiotic treatment, and correlation with microbiological status at start of treatment.

	Microbiological results at start of treatment			
Clinical outcome at 4 week assessment	Ziehl-Neelsen staining		Culture	
	ZN+ (%)	ZN- (%)	Culture+ (%)	Culture- (%)
Treatment failure (n = 41)	36 (75.0)	5 (38.5)	19 (86.4)	22 (56.4)
Status quo (n = 11)	9 (18.6)	2 (15.4)	2 (9)	9 (23.0)
Success (n = 9)	3 (6.3)	6 (46.2)	1 (4.5)	8 (20.5)
TOTAL	48 (100.0%)	13(100.0%)	22(100.0%)	39(100.0%)

Status quo = no change in lesions.

All patients underwent surgical excision after the 4th week except one PCR negative patient who refused surgery. This patient died one month after the end of treatment due to septicemia. After surgical excision of the lesions in the 91 remaining patients, skin grafting was performed when good granulation tissue had formed.

Table 2 presents the clinical outcome at the end of the 12th week of treatment. All but one patient in group I were cured (98.4% success). The failed case developed disseminated BU with osteomyelitis. For patients in group II, 25 patients (83.3%) were cured; lesions of 4 (13.3%) patients deteriorated and one patient remained unchanged. The difference in the outcome at 12 week between group I and group II was statistically significant (p = 0.023). In addition, there was a significant difference (p = 0.026) in the average time of scarring of ulcers between group I and group II patients. Indeed, PCR positive patients had a longer average time to scarring (10.4 weeks) than PCR negative patients (7.5 weeks). The 4 failure cases were treated with regular dressings only and were cured after seven to 12 months.

Two recurrences were observed among the 61 patients with positive PCR. A 7-year-old patient presented with new ulceration at the original site five months after the ulcer had healed. Microbiologic analyses (ZN and PCR) of biopsy specimens were negative. After interview, it became clear that the patient's scar had been accidentally traumatized. The lesion completely healed after a few days and thus, this was not a true recurrence. An 8-year-old patient presented with an ulcer associated with osteomyelitis of the humerus at the original site of the lesion (right elbow) six months after the end of R+S treatment. This patient also showed functional limitations with contracture and substantial decrease of mobility in the right elbow. No laboratory test was performed at recurrence as the patient and his parents refused surgical biopsy and any surgical prevention of disability treatment at this time. Seven months after the recurrence, the patient accepted surgery but despite interventions performed by plastic surgeons, the patient was left with severe sequelae.

No disease recurrence was observed among patients with negative PCR.

## Discussion

This cohort study of large BU-like lesions in rural RDC has two major outcomes: i) the positive predictive value of the WHO clinical case definition (i.e. the number of true BU cases among all the BU-like lesions studied) was low, and ii) delaying surgical treatment to week 4 of antibiotic treatment may be detrimental for ZN positive cases with large ulcers. The criteria for case ascertainment used in this study to discern whom to treat for BU were primarily clinical and epidemiological. We analyzed clinical outcomes of the patients in our cohort into two distinct groups according to PCR results. PCR analysis was retained because of its high sensitivity compared to the other laboratory tests for the diagnostic confirmation of BU [20]–[21]. Using a clinical diagnosis as reference standard, Chauty et al. [12] obtained a PCR positivity of 57.2%. Using the same reference standard, Mensah-Quainoo et al. [22] had a PCR positivity of 72.3% and Stienstra et al. [23] had 74.8%. In our study, the PCR positivity was 66.3% and did not substantially differ from the above mentioned publications in which clinical diagnosis was the only reference standard.

The fact that 33.7% (31/92) of our clinically suspected cases of BU were PCR negative raises the question of whether or not these ulcers were really *M. ulcerans* infections. Clinically suspected cases of BU may be PCR negative if the collection of specimens is not adequate. In our study, the collected specimens were inadequate for histopathologic diagnosis of BU for 5 of 29 PCR negative patients (biopsy specimens too superficial). It is, however, unlikely that the PCR negative results were related to inadequate sampling because for each patient, 2 to 5 specimens were collected and other tests (ZN stained smears and culture) were negative for all specimens.

Among the 29 PCR negative patients analyzed by histopathology, 25 showed histopathologic features not compatible with BU at start of treatment. Most specimens showed chronic inflammation and some showed bacterial infection due to gram positive cocci. Microbiologic and histopathologic analyses indicate that the PCR negative patients were most likely not BU cases although the clinical aspects of the ulcerated lesions were considered compatible with BU by three physicians who made the diagnosis in DRC before treatment. The histopathologic examinations provided accurate diagnoses for some of these cases (ulcers due to bacterial infections with gram positive cocci, vascular disorders, dermatophytosis) [24], [25], [26].

Four PCR negative patients, however, showed histopathologic features compatible with BU (extensive coagulation necrosis in subcutis) but no AFB were seen. Their clinical status at 4 weeks was deteriorating. Histopathologic changes of these lesions were nonspecific [17], [25], [27]. In our opinion, the absence of AFB in histologic examination and negative PCR results make the diagnosis of BU very unlikely for most patients classified in group II, thus we may conclude that the positive predictive value of a clinical case definition of BU was low in this series of large ulcerated lesions.

A post-hoc confirmation of the clinical diagnosis of a sample of PCR negative patients, based on retrospective examination of photographs taken before treatment, did not reveal typical BU features.

The clinical diagnosis of ulcerated forms of BU may be more difficult than is usually recognized, underlining the importance of confirmation by laboratory tests [1], [20]. The clinical response after 4 weeks of antibiotic treatment was significantly more successful for PCR negative patients (54.8%) than for PCR positive patients (14.8%). If these PCR negative ulcers were due to bacteria other than *M. ulcerans* it is likely that the antibiotic treatment was efficient against these bacteria (gram positive coccal infections).

For PCR positive patients, the clinical outcome after 4 weeks was related to the ZN positivity at the start of antibiotic treatment. Indeed, successful treatment after 4 weeks of antibiotic treatment was observed in 46.2% (6/13) of the ZN negative patients and in 6.3% (3/48) of the patients who were ZN positive at the start of treatment. Deterioration of the ulcers was observed in 87.8% (36/41) of the ZN positive patients and in 12.2% (5/41) of the ZN negative patients.

Increase in size of ulcer after 4 weeks, however, does not necessarily imply treatment failure. Paradoxical worsening during treatment was recently reported by O'Brien et al. [28]. As stated by Johnson [29], these reactions may indeed “contribute to the view that antibiotics are ineffective”. According to Chauty et al. [30], Nienhuis et al. [31] and O'Brien et al. [28], these reactions may be characterized by an initial clinical improvement on antibiotic treatment followed by clinical deterioration and by symptoms such as pain and increasing local temperature. Accordingly, histopathologic examination of excised tissue after antibiotic treatment shows florid inflammatory reactions [28].

None of our patients whose lesions enlarged after 4 weeks of antibiotic treatment presented an initial improvement during the first weeks of antibiotherapy or experienced pain or increased local temperature. Moreover, histopathologic examination of tissue excised after 4 weeks of treatment only revealed an increase of the chronic type of inflammatory response in some patients, as previously described following antibiotic treatment [29]. A significant decrease of the positivity for the laboratory tests was, however, observed after 4 weeks of antibiotic treatment indicating that the drugs had some effect on the bacilli (data not shown). Loss of potency of the antibiotics was not an issue since cold chain measures were respected and antibiotics were kept in refrigerators.

We, therefore, consider these clinical deteriorations after 4 weeks as probable failures. Although paradoxical reactions during antibiotic treatment should be better documented in patients with large ulcerated lesions, we believe that ZN positive patients should be treated by surgery without delay since previous studies have suggested an association between the ZN positivity of cutaneous lesions and bone dissemination [32]. This concern was illustrated by one of our PCR positive patients who was ZN positive and did not present any clinical evidence of bone involvement at the start of treatment but developed a recurrence with osteomyelitis and severe deformities six months after the end of antibiotic treatment. The need of immediate surgery for ZN positive large ulcers remains, however, speculative and further studies are required to determine its importance in the management of BU.

After 12 weeks of antibiotic treatment including surgery after the 4th week, 98.4% (60/61) of the PCR positive patients and 83.3% (25/29) of the PCR negative patients were cured. The 16.7% of PCR negative patients who were not cured in our series could have been cases of non-bacterial origin.

After a follow-up of 2 years, there was only one recurrence among the 91 patients (1.1%). This recurrence rate falls within the range of <2% published by WHO [1]. Indeed, according to WHO, recurrences, reported in 16–30% of cases after surgical treatment alone, have fallen to <2% following the introduction of antibiotics (R+S) [1]. Given our high cure (92.4%) and low recurrence rates (1.1%), it seems beneficial to treat large ulcers, whether BU confirmed or not, with antibiotics. The very low relapse rate confirms the efficacy of antibiotics. However, the need and the best time for surgery for large ulcers should be clarified. Further studies are also required to define the type of antibiotic therapy for non-BU large ulcers, and ideally should be based on specific diagnoses.

A potential weakness of our study is lack of information on the HIV status of our patients, but at the time of the study there was not yet any regular HIV counseling nor antiretroviral care available. The prevalence of HIV infection in the rural area of Nsona Mpangu is less than 3.0% according to the “Programme National Multisectoriel de Lutte contre le VIH/SIDA” [33]. Co-infection with HIV should however be studied in DRC and elsewhere. In Benin, a case-control study comparing HIV-1/HIV2 seroprevalence in BU patients suggests HIV seropositivity increases the risk for BU [34]. HIV infection may also render BU highly aggressive, especially with regard to osteomyelitis. There is also an urgent need for studies to evaluate treatment of HIV positive BU patients with R+S and antiretroviral drugs [35].

The strengths of this study are that: 1) the study was performed in a remote rural BU endemic area; 2) for the first time the antibiotic treatment of patients with large ulcerated lesions was documented with a follow-up of at least two years; 3) all cases were laboratory confirmed by several tests including histology.

In conclusion, our study shows that health professionals dealing with BU may have difficulties in recognizing large ulcers due to *M. ulcerans* on clinical and epidemiologic basis only, hence the importance of microbiologic confirmation by ZN staining at rural health centres. Furthermore, in ZN positive large ulcerated forms of BU (≥ to 10 cm in longest diameter), the efficacy of antibiotic treatment recommended by the WHO should be better documented and the need and the best time for surgery must be clarified. Finally, our data show that it is possible to successfully treat 92.4% (85/92) of patients suffering from large ulcers (whether due to *M. ulcerans* or not) with low recurrence rates (1.1%) by combining an antibiotic treatment with surgery in a rural zone. The data also highlight the need for more specific management of non-BU ulcers.

## Supporting Information

Alternative Language Abstract S1 Translation of the abstract into French by Kapay Kibadi, Marleen Boelaert, and Françoise Portaels.
(0.03 MB DOC)

Alternative Language Abstract S2 Translation of the abstract into Spanish by Marleen Boelaert.
(0.03 MB DOC)

Checklist S1 STROBE Checklist.
(0.10 MB DOC)
