# Supplementary material for: Response to Treatment in a Prospective Cohort of Patients with Large Ulcerated Lesions Suspected to Be Buruli Ulcer (Mycobacterium ulcerans Disease)
Source: PLoS Negl Trop Dis. 2010 Jul 6;4(7):e736. doi: 10.1371/journal.pntd.0000736 (PMC2897843; doi:10.1371/journal.pntd.0000736)
Supplement: Alternative Language Abstract S1 — Translation of the abstract into French by Kapay Kibadi, Marleen Boelaert, and Françoise Portaels. (0.03 MB DOC) [file pntd.0000736.s001.doc]

**Etude prospective d’une cohorte de patients avec des larges ulcères suspects d’ulcère de Buruli (infection à *Mycobacterium ulcerans*) : réponse au traitement**.

**Résumé**

Introduction

L’Organisation Mondiale de la Santé (OMS) recommande la combinaison rifampicine (R) - streptomycine (S) associée ou non à la chirurgie pour le traitement de l’ulcère de Buruli (UB) (infection à *Mycobacterium ulcerans*). Dans des zones endémiques, les cas sont identifiés sur base d’une définition clinique. Nous avons évalué l’efficacité de ce traitement sur une série de patients avec larges ulcères (≥ 10 cm de grand diamètre), dans une zone rurale en République Démocratique du Congo.

Méthodologie

Une cohorte de 92 patients avec de larges ulcères suspects d’UB a été enrôlée d’octobre 2006 à septembre 2007, et traitée selon les recommandations de l’OMS. Chez tous ces patients, des analyses microbiologiques (examen direct selon la méthode de ZIEHL-NEELSEN [ZN], culture et PCR) ont été effectuées. Des analyses histopathologiques ont été également effectuées. Tous les patients on été traités par chirurgie après 4 semaines d’antibiothérapie. Le traitement supervisé avec R-S a été administré quotidiennement pendant 12 semaines, sous observation directe. Les patients ont été suivis deux ans après la fin du traitement.

Résultats

Parmi ces 92 patients, 61 étaient positifs pour *M. ulcerans* par PCR et 31 patients étaient PCR négatifs. Les patients à PCR négative ont connu une meilleure évolution clinique après 4 semaines d’antibiotiques seuls que les patients à PCR positive (54,8 versus 14,8 %). Pour les patients à PCR positive, les résultats du traitement médical après 4 semaines d’antibiothérapie, sont en rapport avec la positivité de l’examen direct réalisé au début du traitement, une détérioration des ulcères étant observée dans 87,8 % (36/41) des patients ZN positifs. L’aggravation de lésions ne semble pas due à des réactions paradoxales. Après la chirurgie effectuée après 4 semaines d’antibiothérapie et 8 semaines additionnelles d’antibiothérapie, 98,4 % de patients à PCR positive et 83,3 % de patients à PCR négative ont été guéris. Un taux de récurrence très bas (1,1 %) a été observé.

Interprétation

La valeur prédictive positive de la définition clinique des cas selon l’OMS est faible. Le faible taux de récurrence confirme l’efficacité des antibiotiques. Néanmoins, pour les ulcères larges, la nécessité d’une intervention chirurgicale et le meilleur moment pour effectuer cette intervention devraient être clarifiés. Nous recommandons qu’un examen direct selon la coloration de ZN soit effectué au niveau des centres de santé ruraux, et que les ulcères larges positifs à l’examen direct soient excisés chirurgicalement sans délai afin d’éviter l’aggravation des lésions.

**Résumé (des auteurs)**

L’ulcère de Buruli (UB) est une malade tropicale négligée dévastatrice causée par *Mycobacterium ulcerans*, avec des séquelles invalidantes en présence de larges lésions nécrosantes non traitées. Les options thérapeutiques sont la chirurgie, les antibiotiques ou une combinaison de deux.

Depuis 2004, l’Organisation Mondiale de la Santé (OMS) recommande les antibiotiques (rifampicine-streptomycine) pour la prise en charge de l’UB. L’efficacité des antibiotiques dans des lésions étendues reste insuffisamment documentée. Nous avons évalué cette stratégie sur des larges ulcères, cliniquement suspects d’UB, dans une zone rurale en République Démocratique du Congo, et avons évalué la valeur du diagnostic clinique.

Tous les patients ont été traités avec les antibiotiques pendant 12 semaines et la chirurgie a été réalisée après 4 semaines. L’UB a été confirmé par des analyses de laboratoires chez 67 % de patients indiquant que le diagnostic clinique de formes ulcérées d’UB est difficile, et cela contrairement à ce qui est souvent rapporté.

Nous recommandons de traiter sans délai avec la chirurgie les cas confirmés positifs à l’examen direct pour éviter que ces lésions ne s’aggravent. Il est possible de traiter avec succès 92 % des patients avec des larges ulcères par la combinaison du traitement antibiotique et de la chirurgie dans une zone rurale et ce avec un faible taux de récurrence (1,1%). Néanmoins, l’indication et le meilleur moment pour réaliser la chirurgie pour des larges ulcères devront être clarifiés.
