# Supplementary material for: Response to Treatment in a Prospective Cohort of Patients with Large Ulcerated Lesions Suspected to Be Buruli Ulcer (Mycobacterium ulcerans Disease)
Source: PLoS Negl Trop Dis. 2010 Jul 6;4(7):e736. doi: 10.1371/journal.pntd.0000736 (PMC2897843; doi:10.1371/journal.pntd.0000736)
Supplement: Alternative Language Abstract S2 — Translation of the abstract into Spanish by Marleen Boelaert. (0.03 MB DOC) [file pntd.0000736.s002.doc]

**Estudio prospectivo de una cohorte de pacientes con úlceras anchas y sospechosas de úlcera de Buruli (infección en *Mycobacterium ulcerans*): respuesta al tratamiento**

**Resumen**

Introducción

La Organización Mundial de la Salud (OMS) recomienda la combinación rifampicine (R) - estreptomicina asociada o no a la cirugía para el tratamiento de la úlcera de Buruli (UB) (infección en *Mycobacterium ulcerans*). En zonas endémicas, los casos son identificados sobre base de una definición clínica. Evaluamos la eficacia de este tratamiento sobre una serie de pacientes con úlceras anchas (≥ 10 cm de gran diámetro), en una zona rural en la República Democrática del Congo.

Metodología

Una cohorte de 92 pacientes con úlceras anchas y sospechosas de UB ha sido enrolada de octubre de 2006 al septiembre de 2007, y tratada según las recomendaciones de la OMS. Las siguientes pruebas microbiológicas han sido efectuados : examen directo según el método de ZIEHL-NEELSEN [ZN], cultura y PCR. Un análisis histopatologica también ha sido efectuado en un sub-grupo. El tratamiento supervisado con R-S ha sido administrado diariamente durante 12 semanas, bajo observancion directa. Todos los pacientes fueron tratados por cirugía después de 4 semanas de antibióticos. Los pacientes han sido seguidos dos años después de fin del tratamiento.

Los resultados

Entre estos 92 pacientes, 61 eran positivos para *M. ulcerans* por PCR y 31 pacientes eran PCR negativo. Los pacientes con PCR negativo conocieron una mejor evolución clínica después de 4 semanas de antibióticos que los pacientes a PCR positivo (54,8 versus el 14,8 %). Para los pacientes con PCR positivo, los resultados del tratamiento antibiotico después de 4 semanas de antibioterapia, estában asociados con la positividad del examen directo realizado al principio del tratamiento. Un deterioro de las úlceras fue observado en el 87,8 % (36/41) de los pacientes ZN positivo. La agravación de lesiones no parecia debida a reacciones paradójicas. Después de la cirugía efectuada después de 4 semanas de antibioterapia y con 8 semanas adicionales de antibioterapia, el 98,4 % de pacientes PCR-positivos y el 83,3 % de pacientes PCR-negativos han sido curados. Una tasa de recurrencia muy baja (1,1 %) ha sido observado.

Interpretación

El valor predictivo positivo de la definición de caso clínica según la OMS es bajo. La tasa de recurrencia baja confirma la eficacia de los antibióticos. Sin embargo, para las úlceras anchas, consta aclarar la necesidad de una intervención quirúrgica y el mejor momento para efectuarla. Recomendamos que un examen directo según la coloración de ZN sea efectuado al nivel de los centros de salud rurales, y que las úlceras anchas positivas al examen directo sean sometidas a la intervencion quirurgica sin demora con el fin de evitar la agravación de las lesiones.

**Resumido por autores**

La úlcera de Buruli (UB) es una enfermedad tropical desatendida devastador causada por *Mycobacterium ulcerans*, con secuelas invalidantes en presencia de lesiones anchas nécrosantes no tratadas. Las opciones terapéuticas son la cirugía, los antibióticos o una combinación de estos.

Desde el 2004, la Organización Mundial de la Salud (OMS) recomienda los antibióticos (rifampicine-estreptomicina) para el manejo del UB. La eficacia de los antibióticos en lesiones extendidas queda poco documentada. Evaluamos esta estrategia sobre úlceras anchas, clínicamente sospechosos de UB, en una zona rural en la República Democrática del Congo, y evaluamos el valor del diagnóstico clínico.

Todos los pacientes han sido tratados con antibióticos durante 12 semanas y la cirugía ha sido realizada después de 4 semanas. El UB ha podido ser confirmado por análisis laboratorial solamente en el 67 % de los pacientes, lo que indica que el diagnóstico clínico de formas ulceradas de UB es difícil, y esto contrariamente a lo que a menudo se afirma.

Recomendamos tratar sin demora con la cirugía los casos confirmados positivos al examen directo para evitar que estas lesiones se agraven. Fue posible tratar con éxito al 92 % de los pacientes con úlceras anchas, por la combinación de un tratamiento antibiótico y de la cirugía en una zona rural y esto, con una tasa de recurrencia baja (1,1 %). Sin embargo, la indicación y el mejor momento para realizar la cirugía para úlceras anchas quedan por aclarar.
